# Supplementary material for: Subclinical thyroid dysfunction and depressive symptoms: protocol for a systematic review and individual participant data meta-analysis of prospective cohort studies
Source: BMJ Open. 2019 Jul 26;9(7):e029716. doi: 10.1136/bmjopen-2019-029716 (PMC6661665; doi:10.1136/bmjopen-2019-029716)
Supplement: Supplementary data [file bmjopen-2019-029716supp001.pdf]

1 Supplementary

1.1 PRISMA-P Checklist

Supplementary Table 1: PRISMA-P (Preferred Reporting Items for Systematic review and Meta-Analysis Protocols) 2015 checklist: recommended items to address in a systematic review protocol*

Section and topic	Item No	Checklist item		Page / Section where item can be found
**ADMINISTRATIVE INFORMATION**				
Title:				
Identification	1a	Identify the report as a protocol of a systematic review	✓	Page 1
Update	1b	If the protocol is for an update of a previous systematic review, identify as such	N.A.	No update
Registration	2	If registered, provide the name of the registry (such as PROSPERO) and registration number	✓	Page 2
Authors:				
Contact	3a	Provide name, institutional affiliation, e-mail address of all protocol authors; provide physical mailing address of corresponding author	✓	Page 1
Contributions	3b	Describe contributions of protocol authors and identify the guarantor of the review	✓	Page 7
Amendments	4	If the protocol represents an amendment of a previously completed or published protocol, identify as such and list changes; otherwise, state plan for documenting important protocol amendments	N.A.	-
Support:				
Sources	5a	Indicate sources of financial or other support for the review	✓	Page 7
Sponsor	5b	Provide name for the review funder and/or sponsor	✓	Page 2
Role of sponsor or funder	5c	Describe roles of funder(s), sponsor(s), and/or institution(s), if any, in developing the protocol	✓	Page 7
**INTRODUCTION**				
Rationale	6	Describe the rationale for the review in the context of what is already known	✓	Page 3
Objectives	7	Provide an explicit statement of the question(s) the review will address with reference to participants, interventions, comparators, and outcomes (PICO)	✓	Page 3
**METHODS**				
Eligibility criteria	8	Specify the study characteristics (such as PICO, study design, setting, time frame) and report characteristics (such as years considered, language, publication status) to be used as criteria for eligibility for the review	✓	Page 4
Information sources	9	Describe all intended information sources (such as electronic databases, contact with study authors, trial registers or other grey literature sources) with planned dates of coverage	✓	Page 5

Search strategy	10	Present draft of search strategy to be used for at least one electronic database, including planned limits, such that it could be repeated	✓	Page 5 , Supplementary
Study records:				
Data management	11a	Describe the mechanism(s) that will be used to manage records and data throughout the review	✓	Page 5
Selection process	11b	State the process that will be used for selecting studies (such as two independent reviewers) through each phase of the review (that is, screening, eligibility and inclusion in meta-analysis)	✓	Page 5
Data collection process	11c	Describe planned method of extracting data from reports (such as piloting forms, done independently, in duplicate), any processes for obtaining and confirming data from investigators	✓	Page 5
Data items	12	List and define all variables for which data will be sought (such as PICO items, funding sources), any pre-planned data assumptions and simplifications	✓	Page 5
Outcomes and prioritization	13	List and define all outcomes for which data will be sought, including prioritization of main and additional outcomes, with rationale	✓	Page 4-5
Risk of bias in individual studies	14	Describe anticipated methods for assessing risk of bias of individual studies, including whether this will be done at the outcome or study level, or both; state how this information will be used in data synthesis	✓	Page 6
Data synthesis	15a	Describe criteria under which study data will be quantitatively synthesised	✓	Page 6-7
	15b	If data are appropriate for quantitative synthesis, describe planned summary measures, methods of handling data and methods of combining data from studies, including any planned exploration of consistency (such as I^2, Kendall's τ)	✓	Page 6-7
	15c	Describe any proposed additional analyses (such as sensitivity or subgroup analyses, meta-regression)	✓	Page 6
	15d	If quantitative synthesis is not appropriate, describe the type of summary planned	N.A.	-
Meta-bias(es)	16	Specify any planned assessment of meta-bias(es) (such as publication bias across studies, selective reporting within studies)	✓	Page 7
Confidence in cumulative evidence	17	Describe how the strength of the body of evidence will be assessed (such as GRADE)	✓	Page 7

*** It is strongly recommended that this checklist be read in conjunction with the PRISMA-P Explanation and Elaboration (cite when available) for important clarification on the items. Amendments to a review protocol should be tracked and dated.** **The copyright for PRISMA-P (including checklist) is held by the PRISMA-P Group and is distributed under a Creative Commons Attribution Licence 4.0.**

N.A.:not applicable

From: Shamseer L, Moher D, Clarke M, Ghersi D, Liberati A, Petticrew M, Shekelle P, Stewart L, PRISMA-P Group. Preferred reporting items for systematic review and meta-analysis protocols (PRISMA-P) 2015: elaboration and explanation. BMJ. 2015 Jan 2;349(jan02 1):g7647.

1.2 Search Strategy

Medline (via Ovid)

1 thyroid diseases/ or hyperthyroidism/ or hypothyroidism/ or thyroid hormones/ or triiodothyronine/ or thyroxine/ or exp Thyrotropin/

2 (subclinical or sub-clinical or mild or subnormal or pre-clinical or preclinical).ti,ab,kw.

3 1 and 2

4 ((subclinical or sub-clinical or mild or subnormal or pre-clinical or preclinical) adj6 (hypothyroid* or hyperthyroid* or thyroid dysfunction* or tri?odothyronin* or thyroxin*or TSH or T4 or T3 or thyroid failure* or thyroid disease*)).ti,ab,kw.

5 ((thyroid function adj6 normal range*) or (TSH adj6 normal range*) or (TSH adj6 range)).ti,ab,kw.

6 4 or 5

7 3 or 6

8 Depression/

9 (depression* or depressive* or depressed* or mood disorder* or (low adj1 mood)).ti,ab,kw.

10 8 or 9

11 7 and 10

12 exp animals/ not humans/

13 11 not 12

Embase (via Ovid)

1 subclinical hypothyroidism/ or subclinical hyperthyroidism/

2 (thyroid disease/ or hyperthyroidism/ or hypothyroidism/ or thyroid hormone/ or thyroxine/ or thyrotropin/) and (subclinical or sub-clinical or mild or subnormal or pre-clinical or preclinical).ti,ab,kw.

3 1 or 2

4 ((subclinical or sub-clinical or mild or subnormal or pre-clinical or preclinical) adj6 (hypothyroid* or hyperthyroid* or thyroid dysfunction* or tri?odothyronin* or thyroxin* or TSH or T4 or T3 or thyroid failure* or thyroid disease*)).ti,ab,kw.

5 ((thyroid function adj6 normal range*) or (TSH adj6 normal range*) or (TSH adj6 range)).ti,ab,kw.

6 4 or 5

7 3 or 6

8 depression/

9 (depression* or depressive* or depressed* or mood disorder* or (low adj1 mood)).).ti,ab,kw.

10 8 or 9

11 7 and 10

12 animal experiment/ not human experiment/

13 11 not 12

CINAHL (via EBSCOhost)

S1 (MH "Thyroid Diseases" OR MH "Hyperthyroidism" OR MH "Hypothyroidism" OR MH "Thyroid Hormones" OR MH "Triiodothyronine" OR MH "Thyrotropin" OR MH "Thyroxine")

S2	(subclinical or sub-clinical or mild or subnormal or pre-clinical or preclinical)
S3	S1 and S2
S4	((subclinical or sub-clinical or mild or subnormal or pre-clinical or preclinical) n6 (hypothyroid* or hyperthyroid* or “thyroid dysfunction*” or tri?odothyronine* or thyroxin* or TSH or T4 or T3 or “thyroid failure*” or “thyroid disease*”))
S5	((“thyroid function*” n6 range*) or (TSH n6 range*) or (TSH n6 range*))
S6	S4 or S5
S7	S3 or S6
S8	MH "Depression"
S9	(depression* or depressive* or depressed* or (“mood disorder*”) or (low n1 mood)).)
S10	S8 or S9
S11	S7 and S10

Cochrane Central (via Wiley)

#1	((subclinical or sub-clinical or mild or subnormal or pre-clinical or preclinical) NEAR/6 (hypothyroid* or hyperthyroid* or thyroid NEXT dysfunction* or tri*odothyronine* or thyroxin* or TSH or T4 or T3 or thyroid NEXT failure* or thyroid NEXT disease*))
#2	((“thyroid function” NEAR/6 range*) or (TSH NEAR/6 range*) or (TSH NEAR/6 range*))
#3	#1 or #2 561
#4	(depression* or depressive* or depressed* or (“mood disorder*”) or (low NEAR/1 mood))
#5	#3 and #4

1.3 Data Request - List of Variables

Supplementary Table 2: Data Request Form: Understanding the Association between Subclinical Thyroid Dysfunction and Depressive Symptoms – an Individual Participant Analysis of Prospective Cohort Studies

**BASELINE:**
Date at Baseline
Thyroid Function:
TSH (mIU/l)
fT4 (ng/dl)
fT3 (pg/ml)
TPO-antibody
Prior depressive Symptoms:
Diagnostic codes or scores to assess depressive symptoms, depressive symptoms at baseline; history
Baseline Diseases:
Dementia (diagnostic codes or scores to assess symptoms of dementia)
History of Diseases:
History of Cancer
History of Cardiovascular Diseases
History of Diabetes
Prior depressive symptoms (diagnostic codes or scores to assess depressive symptoms, depressive symptoms at baseline; history)
Medication:
Medication use at baseline and follow-up: thyroid-altering medications (including thyroxine, anti-thyroid medication, lithium, amiodarone, glucocorticoids, iodine, aspirin, furosemide), antidepressant medications (e.g. SSRIs), preferably at same time as depressive symptoms were assessed.
Other:
Sex
Age
Date of birth (if full date is unavailable please give year of birth)
Physical Activity
Ethnicity
Education (years of school)
Income
Smoking status (never, former, current)
Alcohol use (yes, no) or (g/day if available)
Height
Weight
BMI
Waist circumference

FOLLOW-UP:

Date of follow-up

Thyroid Function:

TSH (mlU/l)

fT4 (ng/dl)

fT3 (pg/ml)

TPO-antibody

Outcome Variable

Primary: depressive symptoms (measured on a standardized scale for all follow-up times available)

Secondary : incidence depression (e.g. ICD-codes)
