# Supplementary material for: Subclinical thyroid dysfunction and depressive symptoms: protocol for a systematic review and individual participant data meta-analysis of prospective cohort studies
Source: BMJ Open. 2019 Jul 26;9(7):e029716. doi: 10.1136/bmjopen-2019-029716 (PMC6661665; doi:10.1136/bmjopen-2019-029716)
Supplement: Author's manuscript [file bmjopen-2019-029716.draft_revisions.pdf]

BMJ Open is committed to open peer review. As part of this commitment we make the peer review history of every article we publish publicly available.

When an article is published we post the peer reviewers' comments and the authors' responses online. We also post the versions of the paper that were used during peer review. These are the versions that the peer review comments apply to.

The versions of the paper that follow are the versions that were submitted during the peer review process. They are not the versions of record or the final published versions. They should not be cited or distributed as the published version of this manuscript.

BMJ Open is an open access journal and the full, final, typeset and author-corrected version of record of the manuscript is available on our site with no access controls, subscription charges or pay-per-view fees (http://bmjopen.bmj.com).

If you have any questions on BMJ Open's open peer review process please email info.bmjopen@bmj.com

BMJ Open

The Association between Subclinical Thyroid Dysfunction and Depressive Symptoms – a Protocol for an Individual Participant Data Analysis of Prospective Cohort Studies

Journal:	*BMJ Open*
Manuscript ID	bmjopen-2019-029716
Article Type:	Protocol
Date Submitted by the Author:	08-Feb-2019

Complete List of Authors:	Wildisen, Lea; University of Bern, Institute of Primary Health Care (BIHAM)<br>Moutzouri, Elisavet; Inselspital, Bern University Hospital, University of Bern, Department of General Internal Medicine; University of Bern, Institute of Primary Health Care (BIHAM)<br>Beglinger, Shanthi; University of Bern, Institute of Primary Health Care (BIHAM); Inselspital, Bern University Hospital, University of Bern, Department of General Internal Medicine<br>Syrogiannouli, Lamprini; University of Bern, Institute of Primary Health Care (BIHAM)<br>Cappola, Anne ; University of Pennsylvania School of Medicine, Division of Endocrinology, Diabetes, and Metabolism, Department of Medicine<br>Asvold, Bjorn; Norwegian University of Science and Technology, K.G. Jebsen Center for Genetic Epidemiology, Department of Public Health and Nursing, NTNU; St. Olavs Hospital, Trondheim University Hospital, Department of Endocrinology<br>Bakker, Stephan; University of Groningen, University Medical Center Groningen, Department of Internal Medicine<br>Ceresini, Graziano; University Hospital of Parma, Department of Clinical and Experimental Medicine, Geriatric Endocrine Unit<br>Dullart, Robin; University of Groningen, Department of Internal Medicine, University Medical Center Groningen<br>Ferrucci, Luigi; Harbor Hospital, Baltimore, National Institute on Aging NIA-ASTRA Unit, Longitudinal Studies Section, Translational Gerontology Branch<br>Grabe, Hans; University Medicine Greifswald, Institute for Community Medicine, Clinical-Epidemiological Research<br>Jukema, J. Wouter; Leiden University Medical Center, Department of Cardiology<br>Nauck, Matthias; University Medicine Greifswald, Institute for Community Medicine, Clinical-Epidemiological Research<br>Trompet, Stella ; Leiden University Medical Center, Section Gerontology and Geriatrics, Department of Internal Medicine<br>Völzke, Henry; University Medicine Greifswald, Institute for Community Medicine, Clinical-Epidemiological Research<br>Westendorp, Rudi; University of Copenhagen, Department of Public Health and Center for Healthy Aging<br>Gussekloo, Jacobijn; Leiden University Medical Center, Department of Public Health and Primary Care; Leiden University Medical Center,

Section Gerontology and Geriatrics, Department of Internal Medicine
Peeters, Robin; Erasmus Medical Center, Department of Medicine
AUJESKY , Drahomir; Inselspital, Bern University Hospital, University of Bern, Department of General Internal Medicine
Bauer, Douglas; University of California, Departments of Medicine and Epidemiology & Biostatistics
Rodondi, Nicolas; University of Bern, Institute of Primary Health Care (BIHAM); Inselspital, Bern University Hospital, University of Bern, Department of General Internal Medicine
Del Giovane, Cinzia; University of Bern, Institute of Primary Health Care (BIHAM)
Feller, Martin; Inselspital, Bern University Hospital, University of Bern, Department of General Internal Medicine; University of Bern, Institute of Primary Health Care (BIHAM)

Keywords: subclinical thyroid dysfunction, subclinical hyperthyroidism, subclinical hypothyroidism, depressive symptoms, individual participant data (IPD), systematic review

SCHOLARONE™
Manuscripts

The Association between Subclinical Thyroid Dysfunction and Depressive Symptoms – a Protocol for an Individual Participant Data Analysis of Prospective Cohort Studies

Lea Wildisen[1], Elisavet Moutzouri[1,2], Shanthi Beglinger[1,2], Lamprini Syrogiannouli[1], Anne R. Cappola[3], Bjørn Åsvold[4,5], Stephan J.L. Bakker[6], Graziano Ceresini[7], Robin P.F. Dullaart[6], Luigi Ferrucci[8], Hans Grabe[9], J. Wouter Jukema[10], Matthias Nauck[9], Stella Trompet[11], Henry Völzke[9], Rudi Westendorp[12], Jacobijn Gussekloo[11,13], Robin Peeters[14], Drahomir Aujesky[2], Douglas Bauer[15], Nicolas Rodondi[1,2], Cinzia Del Giovane[1] & Martin Feller[1,2] for the Thyroid Studies Collaboration.

Author Affiliations

[1]Institute of Primary Health Care (BIHAM), University of Bern, Bern, Switzerland;

[2]Department of General Internal Medicine, Inselspital, Bern University Hospital, University of Bern, Bern, Switzerland;

[3]Division of Endocrinology, Diabetes, and Metabolism, Department of Medicine, University of Pennsylvania School of Medicine, Philadelphia, Pennsylvania, United States;

[4]K.G. Jebsen Center for Genetic Epidemiology, Department of Public Health and Nursing, NTNU, Norwegian University of Science and Technology, Trondheim, Norway;

[5]Department of Endocrinology, St. Olavs Hospital, Trondheim University Hospital, Trondheim, Norway

[6]Department of Internal Medicine, University Medical Center Groningen, University of Groningen, Groningen, the Netherlands;

[7]Department of Clinical and Experimental Medicine, Geriatric Endocrine Unit, University Hospital of Parma, Parma, Italy;

[8]Longitudinal Studies Section, Translational Gerontology Branch, National Institute on Aging, Baltimore, Maryland, United States;

[9]Institute for Community Medicine, Clinical-Epidemiological Research, University Medicine Greifswald, Greifswald, Germany;

[10]Department of Cardiology, Leiden University Medical Center, Leiden, the Netherlands

[11] Section Gerontology and Geriatrics, Department of Internal Medicine, Leiden University Medical Center, Leiden, the Netherlands;

[12]Department of Public Health and Center for Healthy Aging, University of Copenhagen, Copenhagen, Denmark;

[13]Department of Public Health and Primary Care, Leiden University Medical Center, Leiden, the Netherlands;

[14]Department of Medicine, Erasmus Medical Center, Rotterdam, the Netherlands;

[15]Departments of Medicine and Epidemiology & Biostatistics, University of California, San Francisco, California, United States.

Corresponding author: Lea Wildisen, lea.wildisen@biham.unibe.ch, Mittelstrasse 43, 3012 Bern, Switzerland, +41 31 631 58 67

Email: Elisavet Moutzouri elisavet.moutzouri@insel.ch, Shanthi Beglinger shanthi.beglinger@extern.insel.ch, Lamprini Syrogiannouli lamprini.syrogiannouli@biham.unibe.ch, Anne Cappola acappola@pennmedicine.upenn.edu, Bjørn Åsvold bjorn.o.asvold@ntnu.no, Stephan J.L. Bakker s.j.l.bakker@umcg.nl, Graziano Ceresini graziano.ceresini@unipr.it, Robin P.F. Dullaart r.p.f.dullaart@umcg.nl, Luigi Ferrucci ferruccilu@grc.nia.nih.gov, Hans Grabe grabeh@uni-greifswald.de, J. Wouter Jukema J.W.Jukema@lumc.nl, Matthias Nauck matthias.nauck@uni-greifswald.de,Stella Trompet S.Trompet@lumc.nl, Henry Völzke voelzke@uni-greifswald.de, Rudi Westendorp westendorp@sund.ku.dk, Jacobijn Gussekloo J.Gussekloo@lumc.nl, Robin Peeters r.peeters@erasmusmc.nl, Drahomir Aujesky drahomir.aujesky@insel.ch, Douglas Bauer DBauer@psg.ucsf.edu, Nicolas Rodondi nicolas.rodondi@biham.unibe.ch, Cinzia Del Giovane cinzia.delgiovane@biham.unibe.ch, Martin Feller martin.feller@insel.ch

Word count: 1931

Key-Words: subclinical hypothyroidism, subclinical hyperthyroidism, subclinical thyroid dysfunction, depressive symptoms, Individual participant data analysis, systematic review.

For peer review only - http://bmjopen.bmj.com/site/about/guidelines.xhtml

Abstract

Introduction: Prospective cohort studies on the association between subclinical thyroid dysfunction and depressive symptoms have yielded conflicting findings, possibly because of differences in age, sex, thyroid-stimulating hormone cut-off levels, or degree of baseline depressive symptoms. Individual participant data (IPD) which encompasses these variables may help clarify this association.

Methods and Analysis: We will conduct a systematic review and individual participant data analysis of prospective studies on the association between thyroid dysfunction and depressive symptoms. We will identify studies through a systematic search of the literature in the EMBASE and MEDLINE databases and from the Thyroid Studies Collaboration (TSC). We will ask the corresponding authors of studies that meet our inclusion criteria to collaborate by providing IPD. Our primary outcome will be depressive symptoms at the first available individual follow-up, measured on a validated depression scale. We will convert all the scores to the Beck Depression Inventory scales (BDI). For each cohort, we will estimate the mean difference of depressive symptoms scores between participants with subclinical hypo- or hyperthyroidism and control adjusted for depressive symptoms scores at baseline. Furthermore, we will adjust our multivariable linear regression analyses for age, sex and length of follow-up. We will pool the effect estimates of all studies in a random-effects meta-analysis. Heterogeneity will be assessed by I^2. Our secondary outcomes will be depressive symptoms at a specific follow-up time, at the last available individual follow-up and incidence depression at the first, last and at a specific follow-up time. For the binary outcome of incidence depression we will use a logistic regression model.

Ethics and dissemination: All patients gave informed consent to participate. Our findings will have considerable implications for practice and patient care, we will seek to publish this systematic review and IPD meta-analysis in a high-impact clinical journal in the relevant field.

Registration: PROSPERO: CRD42018091627

For peer review only

Strengths and Limitation of this Study

- This will be the first systematic review and meta-analysis with individual participant data (IPD) to assess the association between subclinical thyroid dysfunction and depressive symptoms.
- By using IPD instead of aggregate data, we can define thyroid dysfunction based on the same criteria across all included studies, which will reduce between-study heterogeneity, and analyse subgroups without ecological fallacy.
- We will investigate the association between subclinical thyroid dysfunction and depressive symptoms in different subgroups (age, sex, prior depressive symptoms and different levels of TSH).

For peer review only - http://bmjopen.bmj.com/site/about/guidelines.xhtml

- A possible limitation of this study could be that studies used different measurement scales to evaluate depressive symptoms, so we will convert all the scores to the Beck Depression Inventory scale to make the scale consistent across studies.

1 Introduction

Mild degrees of thyroid dysfunction are common in the general population, especially amongst the elderly [1]. Patients are usually diagnosed with subclinical thyroid dysfunction if their serum thyroid-stimulating hormone (TSH) levels are either abnormally high or low, but their peripheral hormones (serum free thyroxine (ft4) for subclinical hypothyroidism or ft4 and tri-iodothyronine (ft3) for subclinical hyperthyroidism) are normal [1]. A subclinical thyroid dysfunction diagnosis may be associated with several negative health outcomes; depression is one of them.

Studies that have explored the association between subclinical thyroid dysfunction and depressive symptoms have yielded conflicting results. Some observational studies report that depressive symptoms were more prevalent in patients with subclinical hypothyroidism (SHypo) than in euthyroid patients [1, 2]. A cross-sectional study that included over 7,500 participants found no association between SHypo and clinically relevant depression, but did find an association between subclinical hyperthyroidism (SHyper) and clinically relevant depressive symptoms [3]. Another cross-sectional study (600 participants) found that neither SHypo nor SHyper were associated with depressive symptoms [4]. The largest prospective study (220,545 participants) reported in the literature to date found no association between SHypo and incidence of depressive symptoms after two years of follow-up [5]. Another prospective study (606 participants) found a significant association between SHyper and depressive symptoms, but not with SHypo [4].

These inconsistencies could be due to differences in participant's age, sex or prior depressive symptoms. By analysing individual participant data from large cohort studies, we can define the influence of age, sex and prior depressive symptoms. Individual participant data analysis is considered to be the best way for synthesising evidence across several studies because it is not subject to ecological fallacy [6].

1.1 Objectives

This systematic review and individual participant data analysis will assess the prospective association between subclinical thyroid dysfunction at baseline and depressive symptoms during follow-up.

2 Methods and Analysis

The protocol adheres to the PRISMA statement for individual participant data systematic reviews and meta-analysis [7] and was registered with the International Prospective Register of Systematic Reviews (PROSPERO) on 15 May, 2018 (registration number: CRD42018091627)

For peer review only - http://bmjopen.bmj.com/site/about/guidelines.xhtml

2.1 Eligibility criteria

Study Design: This systematic review will include prospective cohort studies that reported data on thyroid function (serum TSH and fT4 if available) at baseline, and prospective follow-up data on depressive symptoms. We will exclude studies that only include depressed participants, only pregnant or postpartum participants, or women who were planning to conceive. We will exclude those studies because we don't want to have depressed and pregnant women overrepresented in our study. This does not mean that we will exclude single depressed and pregnant individuals from studies.

Participants: We will include participants 18 years and older. We will exclude participants with overt hypo- or hyperthyroidism from the analysis, as most indications are clear for treatment of these conditions.

Exposed and Control Group: The exposed group will be participants with SHyper or SHypo. We define SHyper as TSH level < 0.45 mIU/L with normal fT4 levels and we define SHypo as TSH levels ≥ 4.5 mIU/L and < 20mIU/L with normal fT4 levels, as in our previous analyses [8, 9]. The range for normal fT4 will be defined as study specific reference ranges, similar to our previous IPD analyses [6, 9]. Participants with missing fT4 but TSH levels within the range of SHypo or SHyper will also be included in the exposed group, because most people with TSH levels in this range have subclinical rather than overt thyroid dysfunction [10]. The control group will be euthyroid participants, defined as having TSH levels between 0.45 to 4.49 mIU/L.

Outcomes: We will consider studies with data on depressive symptoms measured on a validated continuous scale (e.g. Beck Depression Inventory, Geriatric Depression Scale, etc.). For the dichotomous outcome we will consider data on incident depressive symptoms among others diagnosed through ICD-10 or DSM-V codes or by established cut-off levels for incidence depressive symptoms on continuous depression scales.

For peer review only

2.2 Outcomes

Our primary outcome will be depressive symptoms measured on a continuous scale at the first individual available follow-up. The secondary outcomes will be depressive symptoms measured on a continuous scale at a specific follow-up time point (e.g. after year 3 follow-up; decision depending on data availability) and at the last available individual follow-up. We will convert all the depressive symptoms scales to the beck depression inventory to make the scale consistence across the study, instead of calculation standardized mean differences for better interpretation of the results. The use of follow-up data as outcome adjusted for baseline instead of the change between follow-up and baseline is recommended for analysis of IPD data, especially if baseline data are balanced between groups [12]. A further secondary outcome will be the incidence of depressive symptoms (binary outcome) at the first follow-up, at a specific-follow-up (e.g. year 3 follow-up) or at the last available individual follow-up.

For peer review only - http://bmjopen.bmj.com/site/about/guidelines.xhtml

2.3 Search Strategy

We will systematically search the literature in the electronic databases MEDLINE and EMBASE. We developed the strategy with the advice of an experienced librarian. We first developed the search for MEDLINE and then we adapted the search for EMBASE (Supplementary). No language restriction will be applied.

We will contact members of the Thyroid Studies Collaboration (TSC, www.thyroid-studies.org), an international network of cohort studies with information on thyroid status, and ask to include their unpublished data.

2.4 Study Selection

We will collect all potential articles identified through the literature search. Multiple publications from the same cohort study will be counted as one study. First, two reviewers (LW and SB) will screen the titles and abstracts and retain articles that meet our eligibility criteria. Second, two reviewers (LW and SB) will determine each remaining article's eligibility by reviewing the full text; the reviewers will keep a detailed record of reasons for excluding studies. We will resolve discrepancies by consensus with a 3rd author (MF).

2.5 Strategy to ask and collect individual participant data

We will use the following strategy to obtain the IPD from each cohort. We will contact the corresponding author of each study by email and ask them to participate in our study by providing individual participant data. If they do not respond, we will email two reminders, including the last and corresponding author. If we still do not get any answer, we will consider the collaboration as declined. If a study will not provide IPD, we will extract aggregate data from the published study and analyse it along with IPD in a sensitivity analysis.

We will send a list of all the variables that we want to include in our analysis to the responsible person from each cohort that agreed to participate (Supplementary). The collaborators will use the list of variables to prepare their datasets and send them to us, or we will download the data from their online database. We will collect these datasets and merge the data into a uniform format (STATA).

2.6 Study quality

We will assess the quality of included studies on the Newcastle-Ottawa Scale (NOS) for cohort studies [11], which allocates a maximum of nine stars to studies of the highest quality based on three parameters: selection of study groups; comparability of groups; and ascertainment of the outcome of interest. Studies will be ranked either high quality (7–9 stars), moderate quality (4–6 stars), or low quality (0–3 stars).

2.7 Data analyses

We will conduct an IPD analysis in two-stages. In the first stage, we will estimate the association between depressive symptoms and participants with subclinical hypothyroidism or subclinical hyperthyroidism and those with normal thyroid function in each cohort with multivariable regression analyses (linear regression for continuous outcomes and logistic regression for dichotomous outcomes), and adjust for depressive symptoms at baseline, length of follow-up, age and sex. For continuous outcomes, we will use the mean difference (MD)

For peer review only - http://bmjopen.bmj.com/site/about/guidelines.xhtml

and its relative 95% confidence interval (CI). For dichotomous outcomes, we will estimate the odds ratio (OR) and its relative 95% CI.

For the continuous outcome, we will transfer all the measurements from different scales to the Beck depression Inventory (BDI) scale by calculating a conversion factor. We will calculate the conversion factor by dividing the range of the BDI scale by the range of the scale we would like to convert. For example, to convert the Center for Epidemiological Studies Depression (CESD) scale to the BDI, we would use a converting factor of 1.01 (63 (BDI range) ÷ 60 (CESD range)). To transfer the measurements from the CESD scale to the BDI each CESD value must be multiplied by the converting factor.

For the dichotomous outcome we will use established cut-off values of the original continuous scales to define depressive symptoms or non-depressive symptoms or we use depressive symptoms diagnosis through ICD-10 or DSM-V codes among others. For example, the CESD score has the best specificity and sensitivity to define incidence depressive symptoms for a cut off value >20 [13]. In the second stage, we will pool the effect measures for each study from the first step using a random effects model.

We will assess the heterogeneity by estimating the τ^2 statistic, the I^2 and the Q-test and explore the source of heterogeneity in several subgroup and sensitivity analyses. If we observe a high heterogeneity across study results we will do a meta-regression including the length of follow-up for each study.

We will perform subgroup analyses by sex, age, TSH levels (Level1: TSH <0.1 mlU/L, Level 2: TSH 0.1-0.44 mlU/L, Level 3: TSH 4.6-6.9 mlU/L, Level 4: 7-9.9 mlU/L, Level 5: TSH≥10 mlU/L), ethnicity (if enough power), prior depressive episodes (yes/no) and thyroxine use at baseline (yes/no).

Our sensitivity analyses will: (1) exclude participants with prior depressive episodes, (2) exclude studies without depressive symptoms data at baseline, (3) exclude participants taking antidepressant medications at baseline or during follow-up, (4) exclude participants with dementia, (5) exclude participants who use thyroxine or anti-thyroid drugs at baseline and follow-up, (6) exclude participants who use thyroid-altering medication (anti-thyroid drugs, thyroxine, amiodarone, lithium), (7) exclude participants without fT4 measured at baseline.

We will assess the presence of publication bias and small study effects with funnel plots and the Egger test [14]. We will use STATA 15v for all analyses.

2.8 Patient and public involvement

Patients were not involved in the development of the research question, outcome measure and study design.

3 Ethics and Dissemination

All patients gave informed consent to participate in these studies. This study may have considerable implications for practice and help improve patient care. This systematic review and IPD meta-analysis will be submitted to a high-impact clinical journal.

For peer review only - http://bmjopen.bmj.com/site/about/guidelines.xhtml

Acknowledgements: The authors thank Beatrice Minder for helping us to develop the literature search strategy and Kali Tal for editing the manuscript.

Authors' contribution: LW and MF drafted the manuscript. All authors reviewed and revised the protocol. CDG and LW designed the statistical methods.

Funding: This systematic review and IPD analysis is funded by a grant from the Swiss National Science foundation (SNSF 320030-172676 to Nicolas Rodondi)

Competing interests: None declared

Ethics approval: This is a protocol paper for analysis of existing cohort data. Each study in the individual participant data set received local ethical approval. We do not plan to recruit further participants and our analysis will not include identifiable data.

For peer review only

For peer review only - http://bmjopen.bmj.com/site/about/guidelines.xhtml

4 References

[1] V. B. Chueire, J. H. Romaldini, and L. S. Ward, "Subclinical hypothyroidism increases the risk for depression in the elderly," *Arch Gerontol Geriatr,* vol. 44, no. 1, pp. 21-8, Jan-Feb 2007.

[2] M. H. Samuels, "Subclinical Hypothyroidism and Depression: Is There a Link?," *Journal of Clinical Endocrinology & Metabolism,* vol. 103, no. 5, pp. 2061-2064, May 2018.

[3] J. W. Hong, J. H. Noh, and D. J. Kim, "Association between subclinical thyroid dysfunction and depressive symptoms in the Korean adult population: The 2014 Korea National Health and Nutrition Examination Survey," (in English), *Plos One,* vol. 13, no. 8, Aug 14 2018.

[4] M. R. Blum *et al.*, "Subclinical Thyroid Dysfunction and Depressive Symptoms among the Elderly: A Prospective Cohort Study," *Neuroendocrinology,* Research Support, Non-U.S. Gov't vol. 103, no. 3-4, pp. 291-9, 2016.

[5] J. S. Kim *et al.*, "Subclinical Hypothyroidism and Incident Depression in Young and Middle-Age Adults," *J Clin Endocrinol Metab,* vol. 103, no. 5, pp. 1827-1833, May 1 2018.

[6] N. Rodondi *et al.*, "Subclinical hypothyroidism and the risk of coronary heart disease and mortality," *JAMA,* vol. 304, no. 12, pp. 1365-74, Sep 22 2010.

[7] L. A. Stewart *et al.*, "Preferred Reporting Items for a Systematic Review and Meta-analysis of Individual Participant Data The PRISMA-IPD Statement," (in English), *Jama-Journal of the American Medical Association,* vol. 313, no. 16, pp. 1657-1665, Apr 28 2015.

[8] M. R. Blum *et al.*, "Subclinical thyroid dysfunction and fracture risk: a meta-analysis," *JAMA,* vol. 313, no. 20, pp. 2055-65, May 26 2015.

[9] C. Baumgartner *et al.*, "Thyroid Function Within the Normal Range, Subclinical Hypothyroidism, and the Risk of Atrial Fibrillation," *Circulation,* vol. 136, no. 22, pp. 2100-2116, Nov 28 2017.

[10] C. Schneider *et al.*, "Initial evaluation of thyroid dysfunction - Are simultaneous TSH and fT4 tests necessary?," *PLoS One,* vol. 13, no. 4, p. e0196631, 2018.

[11] X. Zeng *et al.*, "The methodological quality assessment tools for preclinical and clinical studies, systematic review and meta-analysis, and clinical practice guideline: a systematic review," *J Evid Based Med,* vol. 8, no. 1, pp. 2-10, Feb 2015.

[12] R. D. Riley *et al.*, "Meta-analysis of randomised trials with a continuous outcome according to baseline imbalance and availability of individual participant data," *Stat Med,* vol. 32, no. 16, pp. 2747-66, Jul 20 2013.

[13] G. Vilagut, C. G. Forero, G. Barbaglia, and J. Alonso, "Screening for Depression in the General Population with the Center for Epidemiologic Studies Depression (CES-D): A Systematic Review with Meta-Analysis," *PLoS One,* vol. 11, no. 5, p. e0155431, 2016.

[14] M. Egger, G. Davey Smith, M. Schneider, and C. Minder, "Bias in meta-analysis detected by a simple, graphical test," *BMJ,* vol. 315, no. 7109, pp. 629-34, Sep 13 1997.

For peer review only

For peer review only - http://bmjopen.bmj.com/site/about/guidelines.xhtml

Supplementary

1.1 Search Strategy

1.1.1 Medline

Database: Ovid MEDLINE(R) and Epub Ahead of Print, In-Process & Other Non-Indexed Citations, Daily and Versions(R) <1946 to November 13, 2018>

Search Strategy:

----

1 thyroid diseases/ or hyperthyroidism/ or hypothyroidism/ or thyroid hormones/ or triiodothyronine/ or thyroxine/ or exp Thyrotropin/ (118706)

2 (subclinical or sub-clinical or mild or subnormal or pre-clinical or preclinical).ti,ab,kw. (448017)

3 Depression/ (104786)

4 (depression* or depressive* or depressed* or mood disorder* or bipolar disorder*).ti,ab,kw. (417161)

5 ((subclinical or sub-clinical or mild or subnormal or pre-clinical or preclinical) adj6 (hypothyroid* or hyperthyroid* or thyroid dysfunction* or tri?odthyronin* or thyroxin* TSH or T4 or T3 or thyroid failure* or thyroid disease*)).ti,ab,kw. (5203)

6 ((thyroid function adj3 normal range*) or (TSH adj3 normal range*) or (TSH adj3 range)).ti,ab,kw. (519)

7 1 and 2 (5800)

8 5 or 6 (5583)

9 7 or 8 (7398)

10 3 or 4 (438415)

11 9 and 10 (311)

12 exp animals/ not humans.mp. [mp=title, abstract, original title, name of substance word, subject heading word, floating sub-heading word, keyword heading word, protocol supplementary concept word, rare disease supplementary concept word, unique identifier, synonyms] (4446823)

13 11 not 12 (286)

1.1.2 Embase

Database: Embase <1974 to 2018 November 08>

Search Strategy: 9.11.2018

----

1 subclinical hypothyroidism/ or subclinical hyperthyroidism/ (3143)

2 (thyroid disease/ or hyperthyroidism/ or hypothyroidism/ or thyroid hormone/ or thyroxine/ or thyrotropin/) and (subclinical or sub-clinical or mild or subnormal or pre-clinical or preclinical).ti,ab,kw. (12693)

3 or/1-2 (13554)

4 ((subclinical or sub-clinical or mild or subnormal or pre-clinical or preclinical) adj6 (hypothyroid* or hyperthyroid* or thyroid dysfunction* or tri?odthyronin* or thyroxin* TSH or T4 or T3 or thyroid failure* or thyroid disease*)).ti,ab,kw. (7762)

5 ((thyroid function adj3 normal range*) or (TSH adj3 normal range*) or (TSH adj3 range)).ti,ab,kw. (905)

6 or/3-5 (14554)

7 depression/ (326055)

8 (depression* or depressive* or depressed* or mood disorder*).ti,ab,kw. (541102)

9 or/7-8 (639286)

10 6 and 9 (854)

11 animal experiment/ not human experiment/ (2276387)

12 10 not 11 (830)

For peer review only

1.2 Data Request - List of Variables

Data Request Form: Understanding the Association between Subclinical Thyroid Dysfunction and Depressive Symptoms – an Individual Participant Analysis of Prospective Cohort Studies

BASELINE:

Date at Baseline

Thyroid Function:

TSH (mIU/l)

fT4 (ng/dl)

fT3 (pg/ml)

TPO-antibody

Prior depressive Symptoms:

Diagnostic codes or scores to assess depressive symptoms, depressive symptoms at baseline; history

Baseline Diseases:

Dementia (diagnostic codes or scores to assess symptoms of dementia)

History of Diseases:

History of Cancer

History of Cardiovascular Diseases

History of Diabetes

Prior depressive symptoms (diagnostic codes or scores to assess depressive symptoms, depressive symptoms at baseline; history)

Medication:

Medication use at baseline and follow-up: thyroid-altering medications (including thyroxine, anti-thyroid medication, lithium, amiodarone, glucocorticoids, iodine, aspirin, furosemide), antidepressant medications (e.g. SSRIs), preferably at same time as depressive symptoms were assessed.

Other:

Sex

Age

Date of birth (if full date is unavailable please give year of birth)

Physical Activity

Ethnicity

Education (years of school)

Smoking status (never, former, current)

Alcohol use (yes, no) or (g/day if available)

Height

Weight

BMI

Waist circumference

For peer review only

**FOLLOW-UP:**
Date of follow-up
Thyroid Function:
TSH (mlU/l)
fT4 (ng/dl)
fT3 (pg/ml)
TPO-antibody
Outcome Variable
Primary: depressive symptoms (measured on a standardized scale for all follow-up times available)
Secondary : incidence depression (e.g. ICD-codes)

For peer review only

BMJ Open

The Association between Subclinical Thyroid Dysfunction and Depressive Symptoms – a Protocol for a Systematic Review and Individual Participant Data Meta-Analysis of Prospective Cohort Studies

Journal:	*BMJ Open*
Manuscript ID	bmjopen-2019-029716.R1
Article Type:	Protocol
Date Submitted by the Author:	03-May-2019

Complete List of Authors:	Wildisen, Lea; University of Bern, Institute of Primary Health Care (BIHAM)<br>Moutzouri, Elisavet; Inselspital, Bern University Hospital, University of Bern, Department of General Internal Medicine; University of Bern, Institute of Primary Health Care (BIHAM)<br>Beglinger, Shanthi; University of Bern, Institute of Primary Health Care (BIHAM); Inselspital, Bern University Hospital, University of Bern, Department of General Internal Medicine<br>Syrogiannouli, Lamprini; University of Bern, Institute of Primary Health Care (BIHAM)<br>Cappola, Anne ; University of Pennsylvania School of Medicine, Division of Endocrinology, Diabetes, and Metabolism, Department of Medicine<br>Asvold, Bjorn; Norwegian University of Science and Technology, K.G. Jebsen Center for Genetic Epidemiology, Department of Public Health and Nursing, NTNU; St. Olavs Hospital, Trondheim University Hospital, Department of Endocrinology<br>Bakker, Stephan; University of Groningen, University Medical Center Groningen, Department of Internal Medicine<br>Ceresini, Graziano; University Hospital of Parma, Department of Clinical and Experimental Medicine, Geriatric Endocrine Unit<br>Dullart, Robin; University of Groningen, Department of Internal Medicine, University Medical Center Groningen<br>Ferrucci, Luigi; Harbor Hospital, Baltimore, National Institute on Aging NIA-ASTRA Unit, Longitudinal Studies Section, Translational Gerontology Branch<br>Grabe, Hans; University Medicine Greifswald, Institute for Community Medicine, Clinical-Epidemiological Research<br>Jukema, J. Wouter; Leiden University Medical Center, Department of Cardiology<br>Nauck, Matthias; University Medicine Greifswald, Institute for Community Medicine, Clinical-Epidemiological Research<br>Trompet, Stella ; Leiden University Medical Center, Section Gerontology and Geriatrics, Department of Internal Medicine<br>Völzke, Henry; University Medicine Greifswald, Institute for Community Medicine, Clinical-Epidemiological Research<br>Westendorp, Rudi; University of Copenhagen, Department of Public Health and Center for Healthy Aging<br>Gussekloo, Jacobijn; Leiden University Medical Center, Department of

	Public Health and Primary Care; Leiden University Medical Center, Section Gerontology and Geriatrics, Department of Internal Medicine<br>Peeters, Robin; Erasmus Medical Center, Department of Medicine<br>Klöppel, Stefan; University of Bern, University Hospital of Old Age Psychatry<br>AUJESKY , Drahomir; Inselspital, Bern University Hospital, University of Bern, Department of General Internal Medicine<br>Bauer, Douglas; University of California, Departments of Medicine and Epidemiology & Biostatistics<br>Rodondi, Nicolas; University of Bern, Institute of Primary Health Care (BIHAM); Inselspital, Bern University Hospital, University of Bern, Department of General Internal Medicine<br>Del Giovane, Cinzia; University of Bern, Institute of Primary Health Care (BIHAM)<br>Feller, Martin; Inselspital, Bern University Hospital, University of Bern, Department of General Internal Medicine; University of Bern, Institute of Primary Health Care (BIHAM)
**Primary Subject Heading**:	Epidemiology
Secondary Subject Heading:	Diabetes and endocrinology, Mental health, Evidence based practice
Keywords:	subclinical thyroid dysfunction, subclinical hyperthyroidism, subclinical hypothyroidism, depressive symptoms, systematic review, individual participant data (IPD) meta-analysis

SCHOLARONE™
Manuscripts

The Association between Subclinical Thyroid Dysfunction and Depressive Symptoms – a Protocol for a Systematic Review and Individual Participant Data Meta-Analysis of Prospective Cohort Studies

Lea Wildisen[1], Elisavet Moutzouri[1,2], Shanthi Beglinger[1,2], Lamprini Syrogiannouli[1], Anne R. Cappola[3], Bjørn Åsvold[4,5], Stephan J.L. Bakker[6], Graziano Ceresini[7], Robin P.F. Dullaart[6], Luigi Ferrucci[8], Hans Grabe[9], J. Wouter Jukema[10], Matthias Nauck[9], Stella Trompet[11], Henry Völzke[9], Rudi Westendorp[12], Jacobijn Gussekloo[11,13], Robin Peeters[14], Stefan Klöppel[15], Drahomir Aujesky[2], Douglas Bauer[16], Nicolas Rodondi[1,2], Cinzia Del Giovane[1], Martin Feller[1,2] for the Thyroid Studies Collaboration.

Author Affiliations

[1]Institute of Primary Health Care (BIHAM), University of Bern, Bern, Switzerland;

[2]Department of General Internal Medicine, Inselspital, Bern University Hospital, University of Bern, Bern, Switzerland;

[3]Division of Endocrinology, Diabetes, and Metabolism, Department of Medicine, University of Pennsylvania School of Medicine, Philadelphia, Pennsylvania, United States;

[4]K.G. Jebsen Center for Genetic Epidemiology, Department of Public Health and Nursing, NTNU, Norwegian University of Science and Technology, Trondheim, Norway;

[5]Department of Endocrinology, St. Olavs Hospital, Trondheim University Hospital, Trondheim, Norway;

[6]Department of Internal Medicine, University Medical Center Groningen, University of Groningen, Groningen, the Netherlands;

[7]Department of Clinical and Experimental Medicine, Geriatric Endocrine Unit, University Hospital of Parma, Parma, Italy;

[8]Longitudinal Studies Section, Translational Gerontology Branch, National Institute on Aging, Baltimore, Maryland, United States;

[9]Institute for Community Medicine, Clinical-Epidemiological Research, University Medicine Greifswald, Greifswald, Germany;

[10]Department of Cardiology, Leiden University Medical Center, Leiden, the Netherlands;

[11] Section Gerontology and Geriatrics, Department of Internal Medicine, Leiden University Medical Center, Leiden, the Netherlands;

[12]Department of Public Health and Center for Healthy Aging, University of Copenhagen, Copenhagen, Denmark;

[13]Department of Public Health and Primary Care, Leiden University Medical Center, Leiden, the Netherlands;

[14]Department of Medicine, Erasmus Medical Center, Rotterdam, the Netherlands;

[15]University Hospital of Old Age Psychiatry, University of Bern, Bern, Switzerland;

[16]Departments of Medicine and Epidemiology & Biostatistics, University of California, San Francisco, California, United States.

Corresponding author: Lea Wildisen, lea.wildisen@biham.unibe.ch, Mittelstrasse 43, 3012 Bern, Switzerland, +41 31 631 58 70

Email: Elisavet Moutzouri elisavet.moutzouri@insel.ch, Shanthi Beglinger shanthi.beglinger@extern.insel.ch, Lamprini Syrogiannouli lamprini.syrogiannouli@biham.unibe.ch, Anne Cappola acappola@pennmedicine.upenn.edu, Bjørn Åsvold bjorn.o.asvold@ntnu.no, Stephan J.L. Bakker s.j.l.bakker@umcg.nl, Graziano Ceresini graziano.ceresini@unipr.it, Robin P.F. Dullaart r.p.f.dullaart@umcg.nl, Luigi Ferrucci ferruccilu@grc.nia.nih.gov, Hans Grabe grabeh@uni-greifswald.de, J. Wouter Jukema J.W.Jukema@lumc.nl, Matthias Nauck matthias.nauck@uni-greifswald.de, Stella Trompet S.Trompet@lumc.nl, Henry Völzke voelzke@uni-greifswald.de, Rudi Westendorp westendorp@sund.ku.dk, Jacobijn Gussekloo J.Gussekloo@lumc.nl, Robin Peeters r.peeters@erasmusmc.nl, Stefan Klöppel stefan.kloeppel@upd.ch, Drahomir Aujesky drahomir.aujesky@insel.ch, Douglas Bauer DBauer@psg.ucsf.edu, Nicolas Rodondi nicolas.rodondi@biham.unibe.ch, Cinzia Del Giovane cinzia.delgiovane@biham.unibe.ch, Martin Feller martin.feller@insel.ch

Word count: 2065

For peer review only

For peer review only - http://bmjopen.bmj.com/site/about/guidelines.xhtml

Key-Words: subclinical hypothyroidism, subclinical hyperthyroidism, subclinical thyroid dysfunction, depressive symptoms, Individual participant data meta-analysis, systematic review.

Abstract

Introduction: Prospective cohort studies on the association between subclinical thyroid dysfunction and depressive symptoms have yielded conflicting findings, possibly because of differences in age, sex, thyroid-stimulating hormone cut-off levels, or degree of baseline depressive symptoms. Analysis of Individual participant data (IPD) may help clarify this association.

Methods and Analysis: We will conduct a systematic review and IPD meta-analysis of prospective studies on the association between thyroid dysfunction and depressive symptoms. We will identify studies through a systematic search of the literature in the Ovid Medline, Ovid Embase, Cochrane CENTRAL, and CINAHL databases from inception to April 2019 and from the Thyroid Studies Collaboration (TSC). We will ask corresponding authors of studies that meet our inclusion criteria to collaborate by providing IPD. Our primary outcome will be depressive symptoms at the first available individual follow-up, measured on a validated scale. We will convert all the scores to the Beck Depression Inventory scale. For each cohort, we will estimate the mean difference of depressive symptoms between participants with subclinical hypo- or hyperthyroidism and control adjusted for depressive symptoms at baseline. Furthermore, we will adjust our multivariable linear regression analyses for age, sex, education, and income. We will pool the effect estimates of all studies in a random-effects meta-analysis. Heterogeneity will be assessed by I^2. Our secondary outcomes will be depressive symptoms at a specific follow-up time, at the last available individual follow-up and incidence depression at the first, last and at a specific follow-up time. For the binary outcome of incidence depression, we will use a logistic regression model.

Ethics and dissemination: Formal ethical approval is not required as primary data will not be collected. Our findings will have considerable implications for patient care. We will seek to publish this systematic review and IPD meta-analysis in a high-impact clinical journal.
PROSPERO: CRD42018091627

Strengths and Limitation of this Study

- This will be the first systematic review and meta-analysis with individual participant data (IPD) to assess the association between subclinical thyroid dysfunction and depressive symptoms.
- By using IPD instead of aggregate data, we can define thyroid dysfunction based on the same criteria across all included studies, which will reduce between-study heterogeneity, and we can analyse subgroups without ecological fallacy.
- We will investigate the association between subclinical thyroid dysfunction and depressive symptoms in different subgroups (age, sex, prior depressive symptoms and different levels of TSH).

For peer review only - http://bmjopen.bmj.com/site/about/guidelines.xhtml

- A possible limitation of this study could be that studies used different scales to measure depressive symptoms, so we will convert all the scores to the Beck Depression Inventory scale to make the scale consistent across studies.

1 Introduction

Mild degrees of thyroid dysfunction are common in the general population, especially amongst the elderly (1). Patients are usually diagnosed with subclinical thyroid dysfunction if their serum thyroid-stimulating hormone (TSH) levels are either abnormally high or low, but their serum free thyroxine (ft4) normal (1) . A subclinical thyroid dysfunction diagnosis may be associated with several negative health outcomes; depression is one of them.

Studies that have explored the association between subclinical thyroid dysfunction and depressive symptoms have yielded conflicting results. Some observational studies report that depressive symptoms were more prevalent in patients with subclinical hypothyroidism (SHypo) than in euthyroid patients (1, 2). A cross-sectional study that included over 7,500 participants found no association between SHypo and clinically relevant depression, but did find an association between subclinical hyperthyroidism (SHyper) and clinically relevant depressive symptoms (3). Another cross-sectional study (606 participants) found that neither SHypo nor SHyper were associated with depressive symptoms (4). The largest prospective study (220,545 participants) reported in the literature to date found no association between SHypo and incidence of depressive symptoms after two years of follow-up (5). Another prospective study (606 participants) found a significant association between SHyper and depressive symptoms, but not with SHypo (4).

These inconsistencies could be due to differences in participant's age, sex or prior depressive symptoms. By analysing IPD from large cohort studies, we can define the influence of age, sex and prior depressive symptoms. IPD meta-analysis is considered to be the best way for synthesising evidence across several studies because it is not subject to ecological fallacy (6).

1.1 Objectives

This systematic review and IPD meta-analysis aims to determine the difference in depressive symptoms between adult participants with SHypo or SHyper and adults with a normal thyroid function (euthyroid participants) during follow-up.

2 Methods and Analysis

The protocol adheres to the PRISMA statement for Individual Participant Data systematic reviews (7) and to the PRISMA statement for systematic review protocols (Checklist Table in Supplementary 1.1). The protocol was

For peer review only

registered with the International Prospective Register of Systematic Reviews (PROSPERO) on 15 May, 2018 (registration number: CRD42018091627).

2.1 Eligibility criteria

We will include studies in any language and any year of publication. We will also consider unpublished studies.
Study Design: This systematic review will include prospective cohort studies that reported data on thyroid function (serum TSH and fT4 if available) at baseline, and prospective follow-up data on depressive symptoms. We will consider any length of follow-up. Case studies or case series (i.e. studies exclusively focusing on participants with thyroid dysfunction) will not be eligible. We will exclude studies that only include depressed participants, only pregnant or postpartum participants, or women who were planning to conceive. We will exclude those studies because we don't want to have depressed or pregnant women overrepresented in our study. This does not mean that we will exclude single depressed or pregnant individuals from studies.
Participants: We will include participants 18 years and older. We will exclude participants with overt hypo- or hyperthyroidism from the analysis, as most indications are clear for treatment of these conditions.
Exposed and Control Group: The exposed group will be participants with SHyper or SHypo. We define SHyper as TSH level < 0.45 mIU/L with normal fT4 levels and we define SHypo as TSH levels ≥ 4.5 mIU/L and < 20mIU/L with normal fT4 levels, as in our previous analyses (8, 9). The range for normal fT4 will be defined as study specific reference ranges, similar to our previous IPD analyses (6, 9). Participants with missing fT4 but TSH levels within the range of SHypo or SHyper will also be included in the exposed group, because most people with TSH levels in this range have subclinical rather than overt thyroid dysfunction (10). The control group will be euthyroid participants, defined as having TSH levels between 0.45 to 4.49 mIU/L.
Outcomes: We will consider studies with data on depressive symptoms measured on a validated continuous scale (e.g. Beck Depression Inventory, Geriatric Depression Scale, etc.). For the dichotomous outcome we will consider data on incident depressive symptoms among others diagnosed through ICD-10 or DSM-V codes or by established cut-off levels for incidence depressive symptoms on continuous depression scales.

2.2 Outcomes

Our primary outcome will be depressive symptoms measured on a continuous scale at the first individual available follow-up. The secondary outcomes will be depressive symptoms measured on a continuous scale at a specific follow-up time point (e.g. after year 3 follow-up; decision depending on data availability) and at the last available individual follow-up. We will convert all the depressive symptoms scales to the beck depression inventory to make the scale consistence across the study for better interpretation of the results. The use of follow-up data as outcome adjusted for baseline instead of the change between follow-up and baseline is recommended for analysis of IPD data, especially if baseline data are balanced between groups (11). A further

For peer review only - http://bmjopen.bmj.com/site/about/guidelines.xhtml
For peer review only

secondary outcome will be the incidence of depressive symptoms (binary outcome) at the first follow-up, at a specific-follow-up (e.g. year 3 follow-up) and at the last available individual follow-up.

2.3 Information Sources and Search Strategy

We will include eligible studies from the Thyroid Studies Collaboration and we will search the Ovid Medline, Ovid Embase, Cochrane CENTRAL and CINAHL electronic databases from inception through April 2019. We developed the search strategy together with two experienced librarians. We developed the search strategies in Ovid Medline and translated it to match the subject headings and keywords for Ovid Embase, Cochrane CENTRAL and CINAHL. The following items were used: thyroid diseases, hyperthyroidism, hypothyroidism, thyroid hormones, triiodothyronine, thyroxine, thyrotropin, subclinical, sub-clinical, mild, subnormal, pre-clinical, preclinical, depression. The details of the search strategy are provided in Supplementary 1.2. No language restriction will be applied.

We will contact members of the Thyroid Studies Collaboration (TSC, www.thyroid-studies.org), an international network of cohort studies with information on thyroid status, and ask to include their unpublished data.

2.4 Study Selection

We will collect all potential articles identified through the literature search. Multiple publications from the same cohort study will be counted as one study. We will export all the identified studies to Endnote X8 and delete duplicates. First, two reviewers (LW and SB) will screen the titles and abstracts and retain articles that meet our eligibility criteria by using a Distiller Systematic Review Software. Second, two reviewers (LW and SB) will determine each remaining article's eligibility by reviewing the full text; the reviewers will keep a detailed record of reasons for excluding studies. We will resolve discrepancies by consensus with a 3rd author (MF).

2.5 Strategy to ask and collect individual participant data

We will use the following strategy to obtain the IPD from each cohort. We will contact the corresponding author of each study by email and ask them to participate in our study by providing IPD. If they do not respond, we will email two reminders, including the last and corresponding author. If we still do not get any answer, we will consider the collaboration as declined. If a study will not provide IPD, we will extract aggregate data from the published study and analyse it along with IPD.

We will distribute a list of all the variables that we want to include in our analysis to the responsible person from each cohort that agreed to participate. The detailed list with all the required variables is provided in the Supplementary 1.3. The collaborators will use the list of variables to prepare their datasets and send them to us, or we will download the data from their online database. We will collect these datasets and merge the data into a uniform format (STATA).

For peer review only - http://bmjopen.bmj.com/site/about/guidelines.xhtml

2.6 Study quality

We will assess the quality of included studies on the Newcastle-Ottawa Scale (NOS) for cohort studies (12), which allocates a maximum of nine stars to studies of the highest quality based on three parameters: selection of study groups; comparability of groups; and ascertainment of the outcome of interest. Studies will be ranked either high quality (7–9 stars), moderate quality (4–6 stars), or low quality (0–3 stars).

2.7 Data analyses

We will conduct an IPD meta-analysis in two-stages. In the first stage, we will estimate the association between depressive symptoms and participants with subclinical hypothyroidism or subclinical hyperthyroidism and those with normal thyroid function in each cohort with multivariable regression analyses (linear regression for continuous outcomes and logistic regression for dichotomous outcomes), and adjust for depressive symptoms at baseline, age, sex, education, and income. For continuous outcomes, we will use the mean difference (MD) and its relative 95% confidence interval (CI). For dichotomous outcomes, we will estimate the odds ratio (OR) and its relative 95% CI.

For the continuous outcome, we will transfer all the measurements from different scales to the Beck depression Inventory (BDI) scale by calculating a conversion factor. We will calculate the conversion factor by dividing the range of the BDI scale by the range of the scale we would like to convert. For example, to convert the Center for Epidemiological Studies Depression (CESD) scale to the BDI, we would use a converting factor of 1.01 (63 (BDI range) ÷ 60 (CESD range)). To transfer the measurements from the CESD scale to the BDI we would each individuals CESD value must multiply by the converting factor.

For the dichotomous outcome we will use established cut-off values of the original continuous scales to define depression or non-depression or we use diagnosis of depression through ICD-10 or DSM-V codes among others. For example, the CESD score has the best specificity and sensitivity to define incidence depressive symptoms for a cut off value >20 (13). In the second stage, we will pool the effect measures for each study from the first step using a random effects model.

We will assess the heterogeneity by estimating the τ^2 statistic, the I^2 and the Q-test and explore the source of heterogeneity in several subgroup and sensitivity analyses. If we observe a high heterogeneity across study results we will do a meta-regression including the length of follow-up for each study.

We will perform subgroup analyses by sex, age (≤57 years, >75 years) , TSH levels (Level1: TSH <0.1 mIU/L, Level 2: TSH 0.1-0.44 mIU/L, Level 3: TSH 4.6-6.9 mIU/L, Level 4: 7-9.9 mIU/L, Level 5: TSH≥10 mIU/L), ethnicity (if enough power), prior depressive episodes (yes/no) and thyroxine use at baseline (yes/no).

Four the primary outcome we will conduct several sensitivity analyses: *(1)* exclude participants with prior depressive episodes, *(2)* include studies without depressive symptoms data at baseline, *(3)* exclude participants taking antidepressant medications at baseline or during follow-up, *(4)* exclude participants with dementia, *(5)* exclude participants who use thyroxine or anti-thyroid drugs at baseline and follow-up, *(6)* exclude participants who use thyroid-altering medication (anti-thyroid drugs, thyroxine, amiodarone, lithium), *(7)* exclude participants without fT4 measured at baseline, *(8)* exclude studies that only provide aggregate data. As further

For peer review only - http://bmjopen.bmj.com/site/about/guidelines.xhtml

sensitivity analysis, *(9)* we will calculate mean differences using the original scale for each study in the first stage and pool the derived standardized mean differences in the second stage.

We will assess the presence of publication bias and small study effects with funnel plots and the Egger test (14). We will use STATA 15v for all analyses. We will use the GRADE approach (www.gradeworkinggroup.org) to assess the confidence on estimates.

2.8 Patient and public involvement

Patients were not involved in the development of the research question, outcome measure and study design.

3 Ethics and Dissemination

Formal ethical approval is not required as primary data will not be collected. This study may have considerable implications for practice and help improve patient care. This systematic review and IPD meta-analysis will be submitted to a high-impact clinical journal.

Acknowledgements: The authors thank Beatrice Minder and Doris Kopp (Institute of Social and Preventive Medicine (ISPM), University of Bern, Switzerland) for helping us to develop the literature search strategy and Kali Tal, PhD (Institute of Primary Health Care (BIHAM), University of Bern, Switzerland) for editing the manuscript. This work was supported in part by the Intramural Research Program at the National Institute on Aging.

Authors' contribution: Wildisen and Feller will have full access to all of the data in the study and take responsibility for the integrity of the data and the accuracy of the data analysis. Wildisen and Feller will have the final responsibility for the decision to submit for publication.

Concept and design: Wildisen, Klöppel, Feller, Del Giovane, Rodondi.

Acquisition, analysis, or interpretation of data: Wildisen, Rodondi, Del Giovane, Feller.

Drafting of the manuscript: Wildisen, Del Giovane, Feller.

Critical revision of the manuscript for important intellectual content: Moutzouri, Beglinger, Syroginannouli, Cappola, Åsvold, Bakker, Ceresini, Dullaart, Ferruci, Grabe, Jukema, Nauck, Trompet, Völzke, Westendorp, Gussekloo, Peeters, Aujesky, Bauer, Rodondi.

Statistical analysis: Wildisen, Syrogiannouli, Del Giovane.

Obtained funding: Rodondi.

Administrative, technical, or material support: Wildisen, Moutzouri, Beglinger, Cappola, Åsvold, Bakker, Ceresini, Dullaart, Ferruci, Grabe, Jukema, Nauck, Trompet, Völzke, Westendorp, Gussekloo, Peeters, Bauer.

For peer review only - http://bmjopen.bmj.com/site/about/guidelines.xhtml

Supervision: Rodondi, Del Giovane, Feller.

Funding: This systematic review and IPD analysis is funded by a grant from the Swiss National Science foundation (SNSF 320030-172676 to Nicolas Rodondi). The Swiss National Science Foundation did not have any role in the design and conduct of the study; collection, management, analysis, and interpretation of the data; or preparation, review, or approval of the manuscript.

Competing interests: None declared

Ethics approval: This is a protocol paper for analysis of existing cohort data. Each study in the individual participant data set received local ethical approval. We do not plan to recruit further participants and our analysis will not include identifiable data.

For peer review only

For peer review only - http://bmjopen.bmj.com/site/about/guidelines.xhtml

4 References

1. Chueire VB, Romaldini JH, Ward LS. Subclinical hypothyroidism increases the risk for depression in the elderly. Arch Gerontol Geriatr. 2007;44(1):21-8.
2. Samuels MH. Subclinical Hypothyroidism and Depression: Is There a Link? J Clin Endocr Metab. 2018;103(5):2061-4.
3. Hong JW, Noh JH, Kim DJ. Association between subclinical thyroid dysfunction and depressive symptoms in the Korean adult population: The 2014 Korea National Health and Nutrition Examination Survey. Plos One. 2018;13(8).
4. Blum MR, Wijsman LW, Virgini VS, Bauer DC, den Elzen WP, Jukema JW, et al. Subclinical Thyroid Dysfunction and Depressive Symptoms among the Elderly: A Prospective Cohort Study. Neuroendocrinology. 2016;103(3-4):291-9.
5. Kim JS, Zhang Y, Chang Y, Ryu S, Guallar E, Shin YC, et al. Subclinical Hypothyroidism and Incident Depression in Young and Middle-Age Adults. J Clin Endocrinol Metab. 2018;103(5):1827-33.
6. Rodondi N, den Elzen WP, Bauer DC, Cappola AR, Razvi S, Walsh JP, et al. Subclinical hypothyroidism and the risk of coronary heart disease and mortality. JAMA. 2010;304(12):1365-74.
7. Stewart LA, Clarke M, Rovers M, Riley RD, Simmonds M, Stewart G, et al. Preferred Reporting Items for a Systematic Review and Meta-analysis of Individual Participant Data The PRISMA-IPD Statement. Jama-J Am Med Assoc. 2015;313(16):1657-65.
8. Blum MR, Bauer DC, Collet TH, Fink HA, Cappola AR, da Costa BR, et al. Subclinical thyroid dysfunction and fracture risk: a meta-analysis. JAMA. 2015;313(20):2055-65.
9. Baumgartner C, da Costa BR, Collet TH, Feller M, Floriani C, Bauer DC, et al. Thyroid Function Within the Normal Range, Subclinical Hypothyroidism, and the Risk of Atrial Fibrillation. Circulation. 2017;136(22):2100-16.
10. Schneider C, Feller M, Bauer DC, Collet TH, da Costa BR, Auer R, et al. Initial evaluation of thyroid dysfunction - Are simultaneous TSH and fT4 tests necessary? PLoS One. 2018;13(4):e0196631.
11. Riley RD, Kauser I, Bland M, Thijs L, Staessen JA, Wang J, et al. Meta-analysis of randomised trials with a continuous outcome according to baseline imbalance and availability of individual participant data. Stat Med. 2013;32(16):2747-66.
12. Zeng X, Zhang Y, Kwong JS, Zhang C, Li S, Sun F, et al. The methodological quality assessment tools for preclinical and clinical studies, systematic review and meta-analysis, and clinical practice guideline: a systematic review. J Evid Based Med. 2015;8(1):2-10.
13. Vilagut G, Forero CG, Barbaglia G, Alonso J. Screening for Depression in the General Population with the Center for Epidemiologic Studies Depression (CES-D): A Systematic Review with Meta-Analysis. PLoS One. 2016;11(5):e0155431.
14. Egger M, Davey Smith G, Schneider M, Minder C. Bias in meta-analysis detected by a simple, graphical test. BMJ. 1997;315(7109):629-34.

For peer review only

For peer review only - http://bmjopen.bmj.com/site/about/guidelines.xhtml

1 Supplementary

1.1 PRISMA-P Checklist

Supplementary Table 1: PRISMA-P (Preferred Reporting Items for Systematic review and Meta-Analysis Protocols) 2015 checklist: recommended items to address in a systematic review protocol*

Section and topic	Item No	Checklist item		Page / Section where item can be found
**ADMINISTRATIVE INFORMATION**				
Title:				
Identification	1a	Identify the report as a protocol of a systematic review	✓	Page 1
Update	1b	If the protocol is for an update of a previous systematic review, identify as such	N.A.	No update
Registration	2	If registered, provide the name of the registry (such as PROSPERO) and registration number	✓	Page 2
Authors:				
Contact	3a	Provide name, institutional affiliation, e-mail address of all protocol authors; provide physical mailing address of corresponding author	✓	Page 1
Contributions	3b	Describe contributions of protocol authors and identify the guarantor of the review	✓	Page 7
Amendments	4	If the protocol represents an amendment of a previously completed or published protocol, identify as such and list changes; otherwise, state plan for documenting important protocol amendments	N.A.	-
Support:				
Sources	5a	Indicate sources of financial or other support for the review	✓	Page 7
Sponsor	5b	Provide name for the review funder and/or sponsor	✓	Page 2
Role of sponsor or funder	5c	Describe roles of funder(s), sponsor(s), and/or institution(s), if any, in developing the protocol	✓	Page 7
**INTRODUCTION**				
Rationale	6	Describe the rationale for the review in the context of what is already known	✓	Page 3
Objectives	7	Provide an explicit statement of the question(s) the review will address with reference to participants, interventions, comparators, and outcomes (PICO)	✓	Page 3
**METHODS**				
Eligibility criteria	8	Specify the study characteristics (such as PICO, study design, setting, time frame) and report characteristics (such as years considered, language, publication status) to be used as criteria for eligibility for the review	✓	Page 4
Information sources	9	Describe all intended information sources (such as electronic databases, contact with study authors, trial registers or other grey literature sources) with planned dates of coverage	✓	Page 5

For peer review only - http://bmjopen.bmj.com/site/about/guidelines.xhtml

Search strategy	10	Present draft of search strategy to be used for at least one electronic database, including planned limits, such that it could be repeated	✓	Page 5 , Supplementary
Study records:				
Data management	11a	Describe the mechanism(s) that will be used to manage records and data throughout the review	✓	Page 5
Selection process	11b	State the process that will be used for selecting studies (such as two independent reviewers) through each phase of the review (that is, screening, eligibility and inclusion in meta-analysis)	✓	Page 5
Data collection process	11c	Describe planned method of extracting data from reports (such as piloting forms, done independently, in duplicate), any processes for obtaining and confirming data from investigators	✓	Page 5
Data items	12	List and define all variables for which data will be sought (such as PICO items, funding sources), any pre-planned data assumptions and simplifications	✓	Page 5
Outcomes and prioritization	13	List and define all outcomes for which data will be sought, including prioritization of main and additional outcomes, with rationale	✓	Page 4-5
Risk of bias in individual studies	14	Describe anticipated methods for assessing risk of bias of individual studies, including whether this will be done at the outcome or study level, or both; state how this information will be used in data synthesis	✓	Page 6
Data synthesis	15a	Describe criteria under which study data will be quantitatively synthesised	✓	Page 6-7
	15b	If data are appropriate for quantitative synthesis, describe planned summary measures, methods of handling data and methods of combining data from studies, including any planned exploration of consistency (such as I^2, Kendall's τ)	✓	Page 6-7
	15c	Describe any proposed additional analyses (such as sensitivity or subgroup analyses, meta-regression)	✓	Page 6
	15d	If quantitative synthesis is not appropriate, describe the type of summary planned	N.A.	-
Meta-bias(es)	16	Specify any planned assessment of meta-bias(es) (such as publication bias across studies, selective reporting within studies)	✓	Page 7
Confidence in cumulative evidence	17	Describe how the strength of the body of evidence will be assessed (such as GRADE)	✓	Page 7

*** It is strongly recommended that this checklist be read in conjunction with the PRISMA-P Explanation and Elaboration (cite when available) for important clarification on the items. Amendments to a review protocol should be tracked and dated. The copyright for PRISMA-P (including checklist) is held by the PRISMA-P Group and is distributed under a Creative Commons Attribution Licence 4.0.**

N.A.:not applicable

From: Shamseer L, Moher D, Clarke M, Ghersi D, Liberati A, Petticrew M, Shekelle P, Stewart L, PRISMA-P Group. Preferred reporting items for systematic review and meta-analysis protocols (PRISMA-P) 2015: elaboration and explanation. BMJ. 2015 Jan 2;349(jan02 1):g7647.

For peer review only - http://bmjopen.bmj.com/site/about/guidelines.xhtml

1.2 Search Strategy

Medline (via Ovid)

1	thyroid diseases/ or hyperthyroidism/ or hypothyroidism/ or thyroid hormones/ or triiodothyronine/ or thyroxine/ or exp Thyrotropin/
2	(subclinical or sub-clinical or mild or subnormal or pre-clinical or preclinical).ti,ab,kw.
3	1 and 2
4	((subclinical or sub-clinical or mild or subnormal or pre-clinical or preclinical) adj6 (hypothyroid* or hyperthyroid* or thyroid dysfunction* or tri?odothyronin* or thyroxin*or TSH or T4 or T3 or thyroid failure* or thyroid disease*)).ti,ab,kw.
5	((thyroid function adj6 normal range*) or (TSH adj6 normal range*) or (TSH adj6 range)).ti,ab,kw.
6	4 or 5
7	3 or 6
8	Depression/
9	(depression* or depressive* or depressed* or mood disorder* or (low adj1 mood)).ti,ab,kw.
10	8 or 9
11	7 and 10
12	exp animals/ not humans/
13	11 not 12

Embase (via Ovid)

1	subclinical hypothyroidism/ or subclinical hyperthyroidism/
2	(thyroid disease/ or hyperthyroidism/ or hypothyroidism/ or thyroid hormone/ or thyroxine/ or thyrotropin/) and (subclinical or sub-clinical or mild or subnormal or pre-clinical or preclinical).ti,ab,kw.
3	1 or 2
4	((subclinical or sub-clinical or mild or subnormal or pre-clinical or preclinical) adj6 (hypothyroid* or hyperthyroid* or thyroid dysfunction* or tri?odothyronin* or thyroxin* or TSH or T4 or T3 or thyroid failure* or thyroid disease*)).ti,ab,kw.
5	((thyroid function adj6 normal range*) or (TSH adj6 normal range*) or (TSH adj6 range)).ti,ab,kw.
6	4 or 5
7	3 or 6
8	depression/
9	(depression* or depressive* or depressed* or mood disorder* or (low adj1 mood)).).ti,ab,kw.
10	8 or 9
11	7 and 10
12	animal experiment/ not human experiment/
13	11 not 12

For peer review only - http://bmjopen.bmj.com/site/about/guidelines.xhtml

CINAHL (via EBSCOhost)

S1 (MH "Thyroid Diseases" OR MH "Hyperthyroidism" OR MH "Hypothyroidism" OR MH "Thyroid Hormones" OR MH "Triiodothyronine" OR MH "Thyrotropin" OR MH "Thyroxine")

S2 (subclinical or sub-clinical or mild or subnormal or pre-clinical or preclinical)

S3 S1 and S2

S4 ((subclinical or sub-clinical or mild or subnormal or pre-clinical or preclinical) n6 (hypothyroid* or hyperthyroid* or “thyroid dysfunction*” or tri?odothyronine* or thyroxin* or TSH or T4 or T3 or “thyroid failure*” or “thyroid disease*”))

S5 ((“thyroid function*” n6 range*) or (TSH n6 range*) or (TSH n6 range*))

S6 S4 or S5

S7 S3 or S6

S8 MH "Depression"

S9 (depression* or depressive* or depressed* or (“mood disorder*”) or (low n1 mood)).)

S10 S8 or S9

S11 S7 and S10

Cochrane Central (via Wiley)

#1 ((subclinical or sub-clinical or mild or subnormal or pre-clinical or preclinical) NEAR/6 (hypothyroid* or hyperthyroid* or thyroid NEXT dysfunction* or tri*odothyronine* or thyroxin* or TSH or T4 or T3 or thyroid NEXT failure* or thyroid NEXT disease*))

#2 ((“thyroid function” NEAR/6 range*) or (TSH NEAR/6 range*) or (TSH NEAR/6 range*))

#3 #1 or #2 561

#4 (depression* or depressive* or depressed* or (“mood disorder*”) or (low NEAR/1 mood))

#5 #3 and #4

For peer review only - http://bmjopen.bmj.com/site/about/guidelines.xhtml

For peer review only

1.3 Data Request - List of Variables

Supplementary Table 2: Data Request Form: Understanding the Association between Subclinical Thyroid Dysfunction and Depressive Symptoms – an Individual Participant Analysis of Prospective Cohort Studies

**BASELINE:**
Date at Baseline
Thyroid Function:
TSH (mIU/l)<br>fT4 (ng/dl)<br>fT3 (pg/ml)<br>TPO-antibody
Prior depressive Symptoms:
Diagnostic codes or scores to assess depressive symptoms, depressive symptoms at baseline; history
Baseline Diseases:
Dementia (diagnostic codes or scores to assess symptoms of dementia)
History of Diseases:
History of Cancer<br>History of Cardiovascular Diseases<br>History of Diabetes<br>Prior depressive symptoms (diagnostic codes or scores to assess depressive symptoms, depressive symptoms at baseline; history)
Medication:
Medication use at baseline and follow-up: thyroid-altering medications (including thyroxine, anti-thyroid medication, lithium, amiodarone, glucocorticoids, iodine, aspirin, furosemide), antidepressant medications (e.g. SSRIs), preferably at same time as depressive symptoms were assessed.
Other:
Sex<br>Age<br>Date of birth (if full date is unavailable please give year of birth)<br>Physical Activity<br>Ethnicity<br>Education (years of school)<br>Income<br>Smoking status (never, former, current)<br>Alcohol use (yes, no) or (g/day if available)<br>Height<br>Weight<br>BMI<br>Waist circumference

For peer review only - http://bmjopen.bmj.com/site/about/guidelines.xhtml

FOLLOW-UP:
Date of follow-up
Thyroid Function:
TSH (mIU/l)
fT4 (ng/dl)
fT3 (pg/ml)
TPO-antibody
Outcome Variable
Primary: depressive symptoms (measured on a standardized scale for all follow-up times available)
Secondary : incidence depression (e.g. ICD-codes)

For peer review only

For peer review only - http://bmjopen.bmj.com/site/about/guidelines.xhtml
